# Investigating the Supercapacitive Performance of Cobalt Sulfide Nanostructures Prepared Using a Hydrothermal Method

**DOI:** 10.3390/ma16134512

**Published:** 2023-06-21

**Authors:** Adil Alshoaibi

**Affiliations:** Department of Physics, College of Science, King Faisal University, P.O. Box 400, Al-Ahsa 31982, Saudi Arabia; adshoaibi@kfu.edu.sa

**Keywords:** cobalt sulfide, hydrothermal method, galvanostatic charge–discharge, specific capacitance

## Abstract

In this study, we synthesized cobalt sulfide (CoS) nanostructures for supercapacitor applications via a one-step hydrothermal method. The effect of hydrothermal temperature on the synthesis process was investigated at temperatures ranging from 160 °C to 220 °C. The structural, morphological, and elemental analyses were performed using X-ray diffraction (XRD), energy-dispersive X-ray spectroscopy (EDX), and scanning electron microscopy (SEM). The XRD patterns show the hexagonal phase of CoS, and the samples prepared at 200 °C have high crystallinity. The samples prepared at other temperatures show amorphousness at lower 2-theta angles. EDX indicated that the sample was of high purity, except that the sample prepared at 220 °C had an additional oxygen peak, indicating that sulfur is not stable at high temperatures. In addition, a cobalt oxide (CoO) peak is also observed in the XRD data of the sample prepared at 220 °C. SEM images show that the particles in the samples prepared at 160 °C and 180 °C are agglomerated due to the high surface energy, whereas the samples prepared at 200 °C and 220 °C have a distinct morphology. Electrochemical analyses such as cyclic voltammetry (CV), electrochemical impedance spectroscopy (EIS), and galvanostatic charge–discharge (GCD) were performed on all samples. The CoS sample prepared at 200 °C exhibited a high specific capacitance (C_sp_) of 1583 F/g at a current density of 1 A/g, with low resistivity and high cycling stability.

## 1. Introduction

Energy consumption has increased as a result of population expansion and the depletion of primary energy sources [1]. Furthermore, primary energy sources have severe environmental consequences such as pollution, global warming, and ozone layer depletion [2,3]. As a result, scientists are working to develop new energy sources that are sustainable, ecologically benign, and defensible. Sources of renewable energy such as wind and solar energy are the greatest solutions for addressing daily energy requirements [4,5,6]. For energy consumption, an uninterruptible power supply is required. Solar energy cannot be used at night, and it will not be windy all day [7]. Therefore, we need this constant supply of energy. Here, storage systems can play an important role in providing uninterrupted energy. Batteries and supercapacitors are the devices that are used for electrical energy storage [8,9,10]. Among these two, the battery has a high energy density while the supercapacitor has a high power density [11,12]. Hence, batteries fail in an application where surge energy is required [13]. A supercapacitor, often known as an electrochemical capacitor, is an electrochemical energy storage device [14]. It can hold up to 100 times the energy per unit mass or volume of conventional capacitors [15]. Electric double-layer capacitors or non-Faraday capacitors store energy through electrostatic ion adsorption and desorption processes, whereas Faraday capacitors store energy through fast reduction and oxidation processes at the electrode–electrolyte interface [16,17]. Supercapacitors outperform batteries in terms of power density, charging rate, and charge/discharge cycles [18]. As a result, supercapacitors can be utilized to replace batteries in situations when high power density, or burst energy, is required [19,20]. Supercapacitors have recently received a lot of interest due to their high power density [21], extended lifespan, and position as a bridge between regular batteries and conventional capacitors [22]. They may also be utilized as a backup power source in conjunction with batteries [23]. Several materials have been prepared for supercapacitor electrodes, such as activated carbon [24], carbon nanotube [25], carbon aerogel [26], conducting polymers [27], metal oxide [28], transition metal oxide [29], and transition metal chalcogenides [30]. Additionally, various techniques have been used for the synthesis of electrode materials such as sol-gel [31], co-precipitation [32], and hydrothermal [33]. CoS is an important transition metal chalcogenide material due to its unique electrochemical [34], electrical [35], and magnetic properties [36]. Its compounds are widely used in energy-storage devices [37], electronic devices [38], hydro-desulfurization [39], and other sectors. CoS is one of the most complicated metal sulfide compounds, with several phases and compositions such as CoS, CoS_2_, Co_1-x_S, Co_2_S_3_, Co_3_S_4_, Co_4_S_3_, and Co_9_S_8_ [40]. CoS provides certain difficulty in regulating morphology due to the variability of stoichiometry [41]. They are caused primarily by the coexistence of reducing oxygen ions and oxidizing sulfide ions, which results in non-stoichiometric CoS [40]. Since cobalt has a high affinity for oxygen, contaminants such as cobalt oxide and cobalt hydroxide are found in the samples of CoS [42]. Another issue is the reaction temperature, which is complicated by CoS’s phase structure [43]. Sangui Lui et al. used the solvothermal method to create long-networked CoS nanotubes [42]. Feng Tao and colleagues developed cobalt sulfide (CoS_x_) for supercapacitor applications. A simple precipitation process was used to obtain cobalt sulfide powders [44]. Houzhao Wan et al. hydrothermally synthesized cobalt sulfide nanotubes for supercapacitor applications [45].

Here, in this study, we prepared CoS nanoparticles through a one-step hydrothermal method at various temperatures, including 160, 180, 200, and 220 °C. Pure CoS with good crystallinity and morphology was obtained. The structural, morphological, elemental, and electrochemical properties of the prepared samples were investigated using XRD, SEM, EDX, CV, GCD, and EIS.

## 2. Experimental

### 2.1. Materials

We have used cobalt nitrate hexahydrate (Co (NO_3_)_2_·6H_2_O), thiourea (CH_4_N_2_S), ethanol (C_2_H_5_OH), methanol (CH_3_OH), and distilled water as precursors for the CoS nanostructure preparations. All the precursors were of high purity (99.99%) and purchased from Sigma Aldrich (St. Louis, MO, USA).

### 2.2. Synthesis

In this study, we employed a one-step hydrothermal method to generate cobalt sulfide (CoS) nanostructures. In one beaker, we mixed 0.95 mg cobalt nitrate hexahydrate as a cobalt source with 45 mL of distilled water and stirred for 30 min at room temperature. Similarly, 0.6 mg of thiourea was added as a sulfur source to the above solution and stirred for 30 min at room temperature. The two solutions were combined and stirred for another 3 h. The mixed solution was then placed in an autoclave and baked in an oven for 12 h. The autoclave was cooled to room temperature after heat treatment. Then, the suspension from the autoclave was removed, and the slurry was washed six times with methanol and distilled water alternately. The resultant final product was dried for 24 h at 80 °C, and the powder was collected for characterization. The balanced chemical reaction between cobalt nitrate hexahydrate and thiourea to form CoS is as follows:

Co(NO_3_)_2_·6H_2_O + 4SC(NH_2_)_2_ → CoS + 2(NH_4_)NO_3_ + 4CO_2_ + 10H_2_O

### 2.3. Physical Characterization

The obtained samples were morphologically and structurally characterized using a scanning electron microscope (SEM) equipped with EDX and powder X-ray diffraction (XRD). The surface morphology of the obtained samples was accomplished using a field emission scanning electron microscope (FESEM, JEOL, JSM-6700F). Energy-dispersive X-ray spectroscopy (EDX) was used in conjunction with the FESEM to analyze the composition and element concentration. The X-ray diffraction (XRD) patterns of the products were recorded at room temperature using a Rigaku D-Max 2400 powder X-ray diffractometer with a copper source of Cu K_α1_ (λ = 1.5418 Å) in a 2-theta configuration.

### 2.4. Electrochemical Characterization

The electrochemical tests were performed in a three-electrode configuration cell containing 1 M KOH as the electrolyte connected to a potentiostat/galvanostat (PG Stat-204). The as-synthesized CoS nanoparticles mixed with nafion were deposited on glassy carbon to serve as the working electrode, with platinum serving as the counter electrode and Ag/AgCl serving as the reference electrode. For preparation of the working electrode, I dissolved 2 mg of active substance in 1 mL of ethanol and then pipetted 1 µL of the solution onto glassy carbon. About 1 µg of CoS nanoparticles were placed on the surface of a glassy carbon electrode. When the glassy carbon electrode had cured, nafion was drop-casted on the electrodes as a binder. The three electrodes were immersed in a 2 molar aqueous solution of potassium hydroxide (KOH) as the electrolyte. The prepared working electrode’s electrochemical performance was evaluated using cyclic voltammetry (CV), galvanostatic charge–discharge (GCD), and electrochemical impedance spectroscopy (EIS).

## 3. Results and Discussion

### 3.1. XRD Analysis of CoS Nanostructures

**Figure 1:**
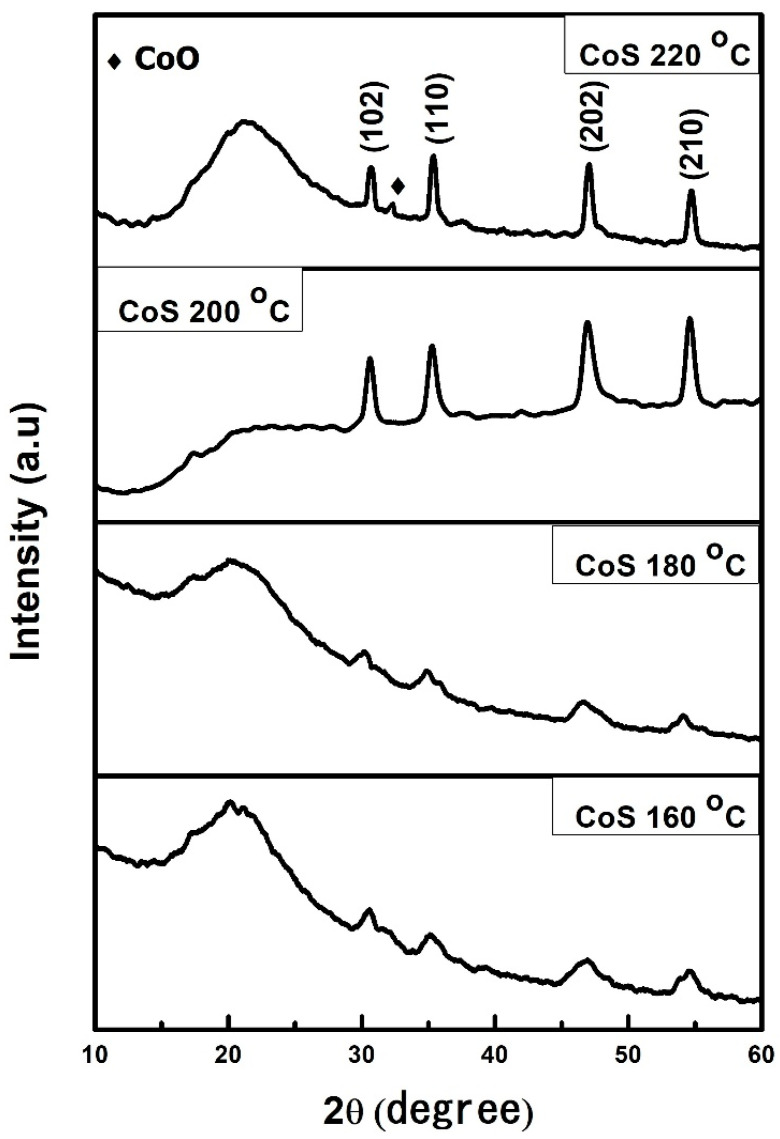
XRD patterns of CoS nanostructures prepared at different hydrothermal temperatures.

To investigate the crystal structure and phase analysis of the prepared CoS samples, XRD was performed using copper as the X-ray source (Cu K_α_, λ = 1.5406 Å) with a 2θ range from 10 to 70 degrees and a step angle of 0.02^0^. The XRD patterns of the CoS nanostructures prepared at different hydrothermal temperatures are shown in Figure 1. The main diffraction peaks at 2θ = 30.64°, 35.28°, 46.94°, and 54.6° match the JCPDS card no. (01-075-0605) and correspond to the (102), (110), (202), and (210) planes of the hexagonal phase of CoS nanostructures. The samples prepared at hydrothermal temperatures of 160 °C, 180 °C, 200 °C, and 220 °C had relatively low-intensity peaks and humps at lower 2-theta angles that indicate the presence of certain amorphous regions within the CoS nanostructures. The lack of complete crystallization or the synthesis of disordered structures is the cause of the amorphousness. In addition, samples prepared at 220 °C have an ultra-low intensity cobalt oxide (CoO) peak, as also confirmed by EDX spectroscopy. This is due to the instability of sulfur and the affinity of cobalt for oxygen at higher temperatures. The samples prepared at 200 °C have better crystallinity and no impurity peaks; therefore, 200 °C is a suitable temperature for the preparation of CoS nanostructures. Furthermore, the average crystallite size is calculated from XRD spectra using Scherrer’s equation, given as

(1) $$D=\frac{k\lambda }{\beta cos\theta }$$

where D is the crystallite size; λ is the wavelength of the X-ray source, i.e., 1.5418 Å; θ is Bragg’s diffraction angle in degree, k is a constant equal to 0.9, and β is the full width at half maxima in radians [46]. Table 1 shows the average crystallite size of the prepared samples. The average crystallite size increases with hydrothermal temperature, from 8 nm for CoS samples prepared at 160 °C to 24 nm for CoS samples prepared at 220 °C.

### 3.2. SEM Analysis of CoS Nanostructures

**Figure 2:**
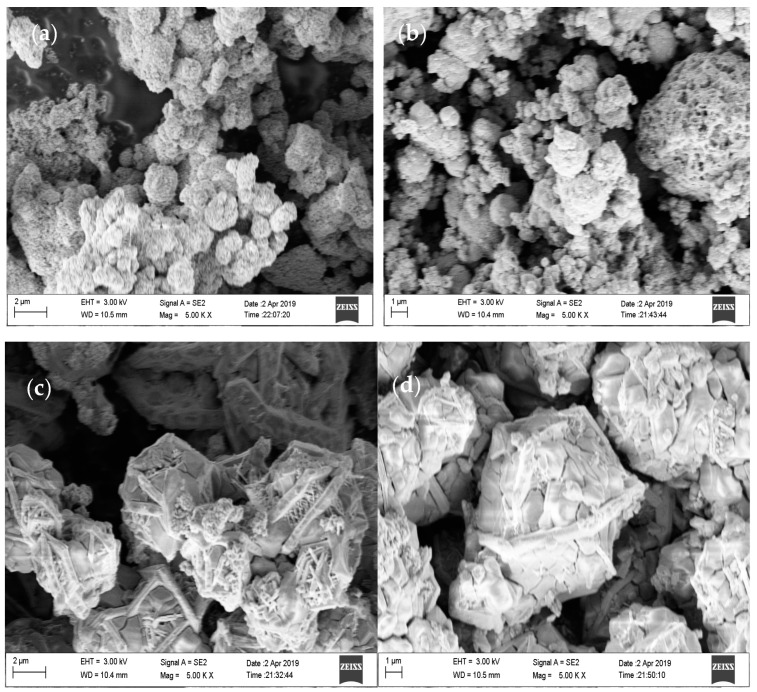
FESEM images of the CoS nanostructures prepared at hydrothermal temperatures of (**a**) 160 °C, (**b**) 180 °C, (**c**) 200 °C, and (**d**) 220 °C.

SEM was used to examine the surface morphology, shape, and particle size of CoS nanoparticles prepared at different hydrothermal temperatures, as shown in Figure 2. The nanoparticles prepared at 160 °C were almost uniform in shape and agglomerated with each other. The agglomeration of nanoparticles may be attributed to the high surface energy of the particles. For the rest of the samples, no uniformity in shape and proper agglomeration was observed. The samples prepared at 180 °C contained nanoparticles of varying sizes. Some of the smaller particles were fused together, but some of them were larger and separated. The samples synthesized at 200 °C were composed of cubes and spherical structures, with mixed grains and boundaries. The samples prepared at 220 °C had spherical structures with a grip around them and well-defined grains and boundaries. The effect of hydrothermal temperature on the morphology, shape, and size of particles can be explained with the help of a kinetic ripening mechanism. At 160 °C, particles had low stability. To reach a steady state, they fused together. When the temperature was raised to 180 °C, some small particles gained enough energy to reach an equilibrium state. Around 200 °C, the precursors gained enough Gibbs free energy to form larger isolated particles. The increase in temperature most likely provided the energy required for the particles to reach an equilibrium state and produce larger and more distinct structures. As the hydrothermal temperature is raised to 220 °C, the particles become clearer, with definite grains and grain boundaries.

### 3.3. EDX Analysis

**Figure 3:**
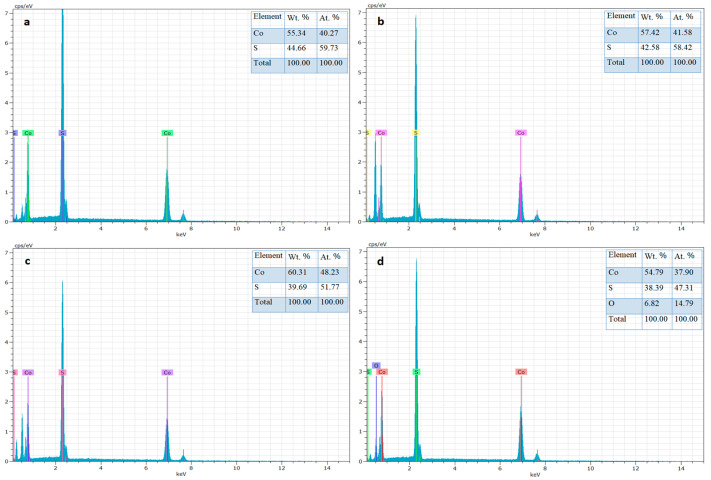
EDX spectra of CoS nanostructures prepared at hydrothermal temperatures of (**a**) 160 °C, (**b**) 180 °C, (**c**) 200 °C, and (**d**) 220 °C.

**Figure 4:**
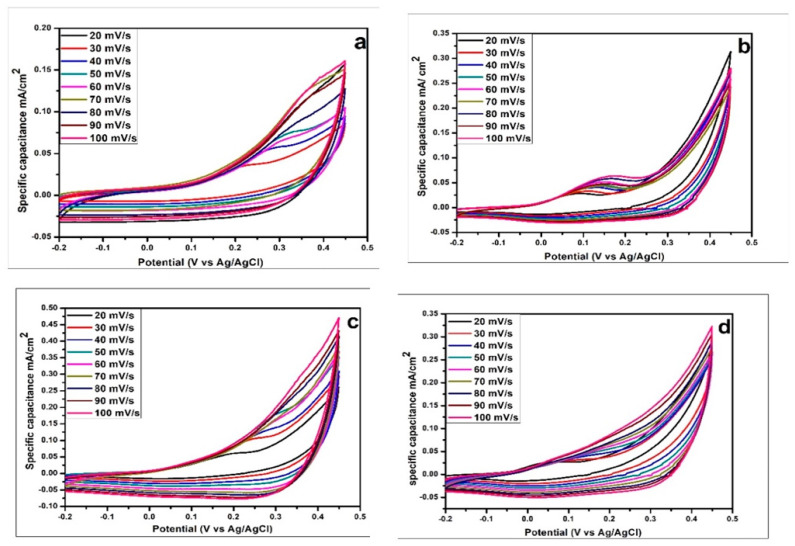
Cyclic voltammetry analysis of CoS nanostructures prepared at hydrothermal temperatures of (**a**) 160 °C, (**b**) 180 °C, (**c**) 200 °C, and (**d**) 220 °C.

For elemental analysis, the energy-dispersive X-ray (EDX) spectroscopy was carried out for the prepared samples shown in Figure 3. The EDX spectra showed only cobalt (Co) and sulfur (S) peaks in all samples, except for the sample with an additional oxygen (O) peak recorded at 220 °C. EDX analysis indicated that the samples were of high purity. However, the oxygen peak observed in the sample prepared at 220 °C is because of the instability of sulfur (S) at a higher temperature. Likewise, the sample prepared at 220 °C hydrothermal temperature showed a decline in the Co:S ratio, confirming the instability of sulfur (S) at higher temperatures. The EDX data are in good agreement with the XRD data. A small peak of CoO was observed in the XRD for the samples prepared at 220 °C. Figure 4 shows the EDX graphs, as well as their weights and atomic percentages.

### 3.4. Electrochemical Analysis

The electrochemical characteristics of CoS nanostructures such as cyclic voltammetry (CV), galvanostatic charge–discharge (GCD), and electrochemical impedance spectroscopy (EIS) were investigated using a potetntiostat/glavanostat.

#### 3.4.1. Cyclic Voltammetry Analysis

**Figure 5:**
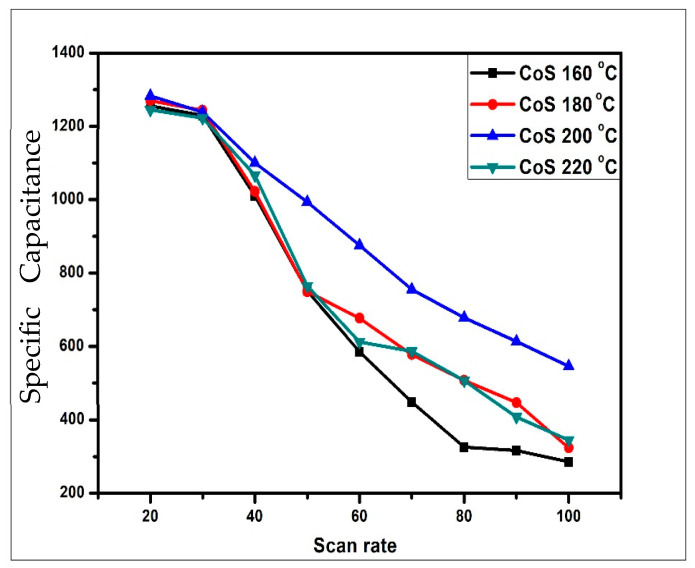
Comparison of specific capacitance C_sp_ with different scan rates.

CV was used to determine the reversibility and nature of the redox reaction in all of the as-prepared CoS samples. The voltage was applied in a cyclic manner and the behavior and value of the current were investigated during each cycle. The electrochemical polarization experiments were carried out in 2 M of a KOH aqueous solution. The CV of CoS samples synthesized at hydrothermal temperatures of 160 °C, 180 °C, 200 °C, and 220 °C was calculated in a potential window ranging from −0.2 to 0.45 V. Figure 4 depicts the cyclic voltammetry curves of CoS nanoparticles at various scan rates ranging from 20 to 100 mV/s. The nearly rectangular shape typical of all CV curves suggests its excellent electrical conductivity and excellent ion transport properties. The four CoS samples (160 °C, 180 °C, 200 °C, and 220 °C) had good linearity of current density with scan rate. The CV curve of an electric double capacitor has an ideal rectangular shape, while a pseudocapacitor has a distorted shape. Figure 5 shows that the CV curve of CoS electrodes has faradic capacitance with a small oxidation peak at different voltages in each of the samples. The specific capacitance C_sp_ of the sample is shown in Table 2 and calculated using Equation [47]

(2) $$C_{s}=\frac{\int_{v1}^{v2}i\left(v\right)dv}{\left(v_{2}-v_{1}\right)vm}$$

where i represents the response of current density to the voltage (V), V_2_ and V_1_ are the upper and lower potential limits, and *v* and m are the scan rate and electrode mass, respectively. The retention percentage is computed as follows:

(3) $$Retention\;\%=\frac{lowest\;C_{sp}}{highest\;C_{sp}}\times 100\%$$

Table 2 and Figure 5 illustrate that as the scan rate increases, the specific capacitance value decreases. The reason is that the high scan rate does not allow electrolyte ions to penetrate to the electrode surface and make greater contact with the inner surface of the electrode material. Therefore, a very small amount of charge is stored on the electrode surface to form an electric double-layer capacitor, and thus, at high scan rates, the specific capacitance decreases in each case. The highest specific capacitance value of 1480 F/g was observed for CoS samples synthesized at 200 °C hydrothermal temperature at a scan rate of 20 mV/s, whereas capacitance values of 1256 F/g, 1270 F/g, and 1245 F/g were obtained for CoS samples synthesized at 160 °C, 180 °C, and 220 °C at the same scan rate.

#### 3.4.2. Electrochemical Impedance Spectroscopy (EIS) Analysis

**Figure 6:**
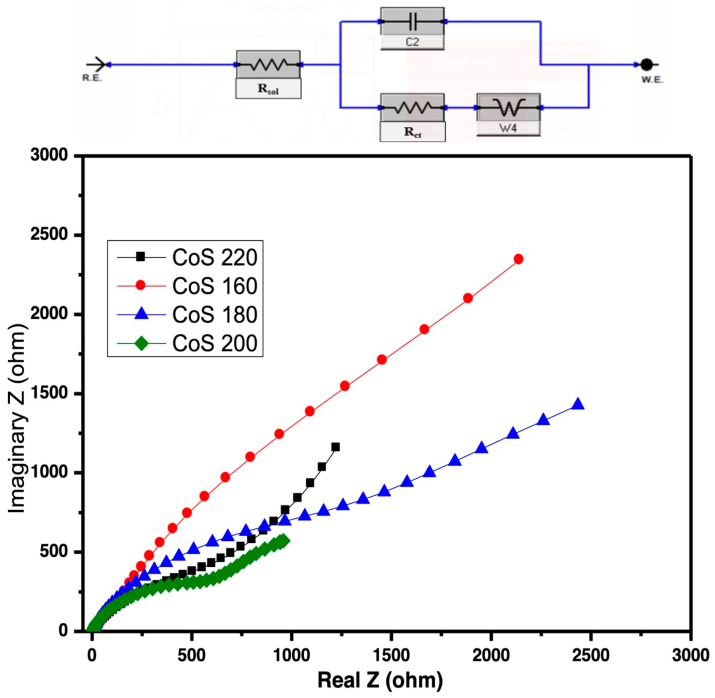
Nyquist plot and the corresponding ESR circuit of the CoS nanostructures prepared at different hydrothermal temperatures.

**Figure 7:**
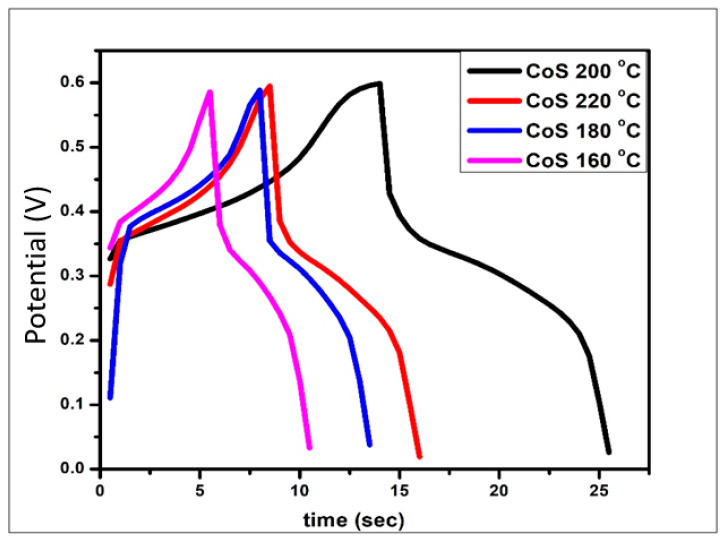
GCD graph of the CoS nanoparticles prepared at various temperatures.

Electrochemical Impedance Spectroscopy (EIS) was used to analyze the surface kinetics and impedance properties of the as-prepared samples. The data are represented as a Nyquist plot in Figure 6. An Equivalent Series Resistance (ESR) circuit was used to simulate its resistance characteristics at low and high frequencies, and the corresponding values are given in Table 3. There are three types of resistance present in the electrochemical cell called solution resistance (R_s_), charge transfer resistance (R_ct_), and Warburg resistance (W). The R_ct_ reflects the electrocatalytic kinetics and can be measured from the diameter of the semi-circle. CoS samples synthesized at 160 °C, 180 °C, and 220 °C had greater R_ct_ values, suggesting slower reaction rates, whereas the sample synthesized at 200 °C had faster reaction rates and lower R_ct_ values. Figure 7 reflects the charge transfer resistance values of each sample, and the R_ct_ value measured for the CoS samples synthesized at 200 °C is about 600 Ohms, which is smaller than the R_ct_ values of CoS samples prepared at 160 °C, 180 °C, and 220 °C. The CoS sample prepared at 200 °C again showed its superior performance due to its smallest R_ct_ value among all samples and the fastest reaction rate. This response might be ascribed to its high crystallinity and distinct morphology.

#### 3.4.3. Galvanostatic Charge Discharge (GCD) Analysis

The charge–discharge ability of the synthesized CoS nanoparticles was evaluated using GCD as shown in Figure 7. When discharging, the potential suddenly drops, which is manifested as electric double-layer capacitance. The plateau of the curve shows the redox reaction that elucidates the pseudocapacitance. The computed specific capacitance values from GCD for CoS samples processed at 160 °C, 180 °C, 200 °C, and 220 °C are 833.3 F/g, 916.6 F/g, 1583.3 F/g, and 116.6 F/g, respectively.

Compared to the existing literature, Smith et al. published specific capacitance values for CoS nanoparticles obtained through a hydrothermal process. Their samples, which were heated to temperatures between 150 °C and 220 °C, displayed specific capacitances between 700 F/g and 1500 F/g, which is consistent with our findings. Similarly, CoS nanosheets made using a different synthesis method were studied by Zhang et al. [27]. When processing samples at 180 °C and 200 °C, respectively, they were able to attain specific capacitance values of roughly 1200 F/g and 1900 F/g. Using a template-assisted technique, Li et al. [42] prepared CoS nanowires. For samples handled at temperatures similar to those in our investigation, their reported specific capacitance values ranged from 800 F/g to 1400 F/g.

**Figure 8:**
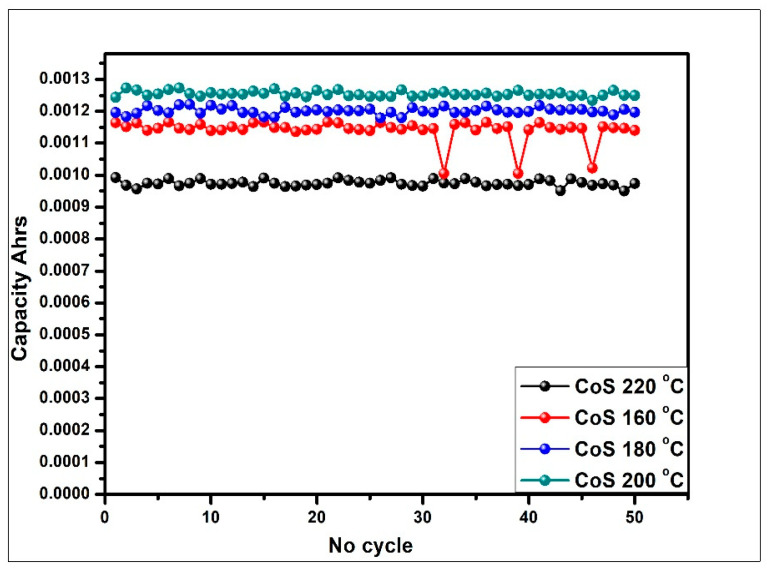
Comparison of cycle number with charge-storage capacity at a current density of 1 A/g for 50 cycles.

The charge storage performance and stability of the CoS samples were also performed with cyclic charge–discharge loading. The graph is drawn to compare the number of cycles with the charge storage capacity, as shown in Figure 8. The investigation was carried out for 50 cycles at a current density of 1 A/g in order to determine the cycling stability of the samples. The CoS sample prepared at 200 °C showed linearity and no deterioration in charge storage capacity, indicating the high stability of the sample.

## 4. Conclusions

In this study, hexagonal-phase cobalt sulfide (CoS) nanostructures were successfully synthesized utilizing a simple one-step hydrothermal technique. XRD, SEM, and EDX were used to characterize the prepared CoS nanoparticles. According to material characterization, the best hydrothermal temperature for the production of cobalt sulfide nanostructures was 200 °C. CoS nanoparticles exhibit poor crystallinity and an amorphous phase below 200 °C, but beyond this temperature, a new phase of cobalt oxide is generated. This implies that sulfur is unstable at higher temperatures, but cobalt has a strong affinity for oxygen. The electrochemical characterizations of CoS reveal its pseudocapacitance behavior. Among all the samples, the sample prepared at 200 °C exhibited the best electrochemical properties, with the highest specific capacitance value of 1583 F/g. The possible reason for the enhanced performance is the optimized crystallinity of the material at this temperature, as evidenced by the XRD analysis. The high crystallinity can enable efficient charge transfer and ion diffusion within the electrode material, leading to improved electrochemical performance.

## Figures and Tables

**Table 1:** Average crystallite size of CoS nanostructures calculated using the Scherrer equation.

S.No:	Sample	Average Crystallite Size
1	CoS 160 °C	8 nm
2	CoS 180 °C	14 nm
3	CoS 200 °C	18 nm
4	CoS 220 °C	24 nm

**Table 2:** Specific capacitance C_sp_ and retention percent calculated from CV data.

Sample	C_sp_ (F/g) 20 mV/s	C_sp_ (F/g) 30 mV/s	C_sp_ (F/g) 40 mV/s	C_sp_ (F/g) 50 mV/s	C_sp_ (F/g) 60 mV/s	C_sp_ (F/g) 70 mV/s	C_sp_ (F/g) 80 mV/s	C_sp_ (F/g) 90 mV/s	C_sp_ (F/g) 100 mV/s	Retention %
CoS 160 °C	1256	1230	1011	752	586	449	326	317	286	21.3
CoS 180 °C	1270	1244	1023	750	677	578	508	447	324	25.5
CoS 200 °C	1480	1239	1101	994	876	756	678	614	546	42.55
CoS 220 °C	1245	1223	1067	765	612	587	507	408	345	27.7

**Table 3:** ESR and the equivalent resistance at lower and higher frequencies of CoS samples.

Sample Name	CoS 160	CoS 180	CoS 200	CoS 220
R_ct_ (ohms)	58	43	10	23
High frequency |Z| (ohms)	87	71	21	22
Low frequency |Z| (ohms)	3173	3224	2479	2993

## Data Availability

Data available in a publicly accessible repository.
